# The Role of Intraoperative and Early Postoperative Blood Pressure Variations, Fluid Balance and Inotropics in Fibula Free Flap Head and Neck Reconstruction: A Retrospective Analysis

**DOI:** 10.3390/jcm12247753

**Published:** 2023-12-18

**Authors:** John-Patrik Burkhard, Alena Wepfer, Lukas M. Löffel, Kaspar F. Bachmann, Patrick Y. Wuethrich

**Affiliations:** 1 Department of Anesthesiology and Pain Medicine, Inselspital, University Hospital Bern, University of Bern, 3010 Bern, Switzerland; 2 Limmat Cleft- and Craniofacial Center Zurich, 8005 Zurich, Switzerland; 3 Department of Anesthesiology, Lindenhof Hospital Bern, 3012 Bern, Switzerland; 4 Department of Intensive Care Medicine, Inselspital, Bern University Hospital, University of Bern, 3010 Bern, Switzerland

**Keywords:** mean arterial pressure, arterial blood pressure, intraoperative hypotension, postoperative complications, systemic complications, free flap surgery, head and neck surgery, fibula free flap, flap complications

## Abstract

Background: In head and neck reconstructive surgery, postoperative complications are a well-known concern. Methods: We examined 46 patients who underwent ablative surgery and received fibula free flap reconstruction. The main focus was to assess the influence of intraoperative blood pressure fluctuations and the administration of inotropic drugs on complications, either related to the flap or systemic, serving as the primary endpoint. Results: Utilizing logistic regression models, we identified that intraoperative mean arterial blood pressure (MAP) drops did not correlate with the occurrence of either flap-related complications (MAP < 70, *p* = 0.79; MAP < 65, *p* = 0.865; MAP < 60, *p* = 0.803; MAP < 55, *p* = 0.937) or systemic medical complications (MAP < 70, *p* = 0.559; MAP < 65, *p* = 0.396; MAP < 60, *p* = 0.211; MAP < 55, *p* = 0.936). The occurrence of flap-related complications significantly increased if a higher dosage of dobutamine was administered (median 27.5 (IQR 0–47.5) vs. 62 (38–109) mg, *p* = 0.019) but not if norepinephrine was administered (*p* = 0.493). This correlation was especially noticeable given the uptick in complications associated with fluid overload (3692 (3101–4388) vs. 4859 (3555–6216) mL, *p* = 0.026). Conclusion: Intraoperative and immediate postoperative blood pressure fluctuations are common but are not directly associated with flap-related complications; however, dobutamine application as well as fluid overload may impact flap-specific complications.

## 1. Introduction

The management of head and neck malignancies through surgical intervention frequently results in considerable defects that necessitate reconstruction using free tissue transfer, aiming to restore function and cosmetic appearance. These surgeries are inherently intricate and carry a notable risk of complications. Thus, it is crucial to identify and address perioperative risk factors to minimize complications and enhance patient outcomes [1].

Special demands on the anesthesiologic care with a focus on maintaining hemodynamic parameters are fundamental and are directed by the administration of intraoperative fluids or vasopressors. However, an increasing number of studies have revealed that the volume of crystalloid fluids administered intraoperatively is a predictor of unfavorable flap outcomes and increased incidence of systemic complications [2,3,4,5,6]. In contrast, there is a prevailing concern among some medical professionals regarding the use of vasopressors due to theoretical fears that they may impair flap perfusion and survival [7]. This historical bias against vasopressor usage originated from studies conducted on animal models [8]. In an effort to avoid the administration of vasopressors or fluid, many patients undergoing free tissue transfer are exposed to episodes of hypotension, particularly at moments of minimal surgical stimulation, such as microvascular anastomoses, which may result in inadequate blood flow and malperfusion to the flap [7,9,10]. Moreover, urinary output, mean arterial pressure, and pulse rate exhibit a circadian rhythm and naturally decrease during the night. This physiological decline is deemed normal and may not necessitate intervention, such as augmenting infusion fluids, to prevent overfilling in patients who have undergone free flap surgery [11].

The impact of the anesthesiologist’s intraoperative management extends beyond the surgical procedure and can have repercussions during the postoperative period. Fluctuations in blood pressure or episodes of hypotension (MAP < 65 mmHg) in non-cardiac surgery are associated with various complications, such as renal insufficiency [12,13], postoperative delirium [14,15], and other significant complications [16,17] following surgeries involving liver transplantation, where large hemodynamic fluctuations are commonly encountered [18].

Given the complex nature of extended surgical procedures, significant intraoperative blood loss is frequently observed and has been associated with a negative outcome [19]. In an effort to mitigate this, anesthesiologists may deliberately maintain hypotension for prolonged periods throughout the procedure. Nevertheless, this strategy can further impede tissue perfusion during both the harvest and reperfusion phases, compromising the success of the free tissue transfer [20]. Currently, the optimal target blood pressure range for this patient population remains uncertain and requires further investigation [5].

This retrospective analysis aimed to examine the correlation between hemodynamic parameters—specifically intraoperative and postoperative blood pressure drops, the use of inotropic agents, and fluid administration—and the incidence of flap-related and systemic complications in a population of patients undergoing head and neck fibula free flap reconstruction surgery.

## 2. Materials and Methods

This retrospective observational study presents a consecutive series of cases conducted at a single tertiary center. The Ethical Committee of the Canton of Bern, Switzerland (KEK-2019-01824) granted ethical approval for this study. The need for informed consent was waived. The study was not registered and adheres to the STROBE (Strengthening the Reporting of Observational Studies in Epidemiology) guidelines.

### 2.1. Study Population

We retrospectively identified 46 consecutive patients who underwent free tissue transfers with fibula flap in the head and neck region between 1 January 2015 and 31 December 2020. This included patients with malignant diseases of the oral cavity, odontogenic tumors, osteoradionecrosis, and osteochemonecrosis of the jaw. All relevant perioperative data were retrieved from the electronic medical record and anesthesia protocols were from the hospital’s internal database.

### 2.2. Data Collection and Outcomes

All perioperative data were collected from the time of surgery until 24 h post operation in the recovery room, until the patient could be transferred to a normal ward. Preoperative data collection recorded gender, age, type of pathology resulting in free tissue transfer with fibula free flap, and comorbidities such as arterial hypertension, chronic obstructive pulmonary disease, diabetes mellitus, renal insufficiency, smoking, and alcohol consumption. Perioperative parameters included blood pressure (systolic, diastolic, and mean arterial pressure (MAP)), total intraoperative administration of IV fluids (crystalloids, colloids, and the number of packed red blood cells), fluid loss, the total dosage of vasopressors (norepinephrine and dopamine) administered, type of postoperative complication, and surgical revisions.

The primary aim was to assess the incidence of flap-specific surgical revisions as well as the occurrence of systemic complications after free tissue transfer in correlation with MAP intraoperatively as well as 24 h postoperatively in the recovery room. Secondly, the administration of inotropic drugs during this period was studied in relation to the occurrence of the above criteria. Revision procedures encompassed all surgical interventions conducted in the operating theatre, whether conducted locally at the recipient site or designed to ensure the survival of the flap. Flap-related complications included flap dehiscence with partial or complete flap necrosis, anastomotic insufficiency, or thrombosis. Our aim was to determine a relationship between intraoperative and postoperative blood pressure drops and flap revisions or possible systemic complications.

### 2.3. Surgical Procedure

The surgical procedure involved a collaborative effort between two teams, with the maxillofacial/ear, nose, and throat (ENT) team handling the ablative surgery, while the plastic surgery and maxillofacial team undertook the flap harvest, micro-anastomoses, and flap inset. To preemptively address potential postoperative airway obstruction, a surgical tracheostomy was performed. Resection, along with neck dissection if indicated, was carried out. Concurrently, fibula free flaps were raised to reconstruct defects in the oral cavity. Once the ablative surgery was completed and the defect reconstruction had immediately commenced, the free flap was detached. A tourniquet was applied to the leg to induce vascular occlusion, with an average pressure of 350 mmHg above the systolic blood pressure maintained throughout the harvesting process, which had a maximum duration of 2 h. This involved the insertion and micro-anastomoses to the neck vessels. The entire surgical procedure adhered to established in-house standards [3].

### 2.4. Measurement and Definition of Intraoperative Blood Pressure Lability and Hypotension

Our anesthetic information system captures systolic and diastolic blood pressure readings through an invasive blood pressure monitor, with MAP values obtained at regular intervals of 5 min. Incomplete records were excluded from the analysis. The MAP value recorded prior to anesthesia induction served as the baseline measurement for each patient. Using linear interpolation, MAP values were then extrapolated to receive measurements minute by minute. Values below a given threshold (e.g., MAP 70, 65, 60, and 55) were then counted and summarized for each patient. Intraoperative and postanesthesia care unit data were collected from the Anesthesia Information System (AIS) (COPRA 6, release 76.5.0; COPRA System GmbH, Berlin, Germany). Arterial blood pressures (systolic blood pressure, mean arterial blood pressure (MAP), diastolic blood pressure) were extracted. Artifacts in the perioperative data were removed for MAP > 155 mmHg or <25 mmHg.

### 2.5. Perioperative Management

The standard monitoring during the procedure included a three-lead ECG, pulse oximetry, and invasive blood pressure measurement through radial artery cannulation. If there was any uncertainty about airway safety, awake fiber optic nasal intubation was performed under continuous administration of remifentanil. The induction medication consisted of propofol (2–3 mg/kg), fentanyl (1–2 μg/kg), and rocuronium (0.6 mg/kg), followed by bladder catheterization. During the initial phase of the surgery, anesthesia was maintained using a combination of propofol and remifentanil until tracheotomy. Subsequently, volatile anesthetics were introduced in combination with dexmedetomidine (0.03–0.05 mg/kg/h) and ketamine (20 mg bolus followed by 0.3 mg/kg/h). These medications were discontinued 30 to 60 min before the end of surgery, along with the resumption of propofol and remifentanil to prevent postoperative nausea and vomiting. Hemodynamic management aimed to maintain a systolic blood pressure of at least 100 mmHg and primarily involved the use of Ringer’s lactate solution. If the perfusion index of the pulse oximetry curve exceeded 5 and urine output ranged between 0.3 and 0.5 mL/kg/h, euvolemia was assumed. In consultation with the lead surgeon, a continuous low-dose administration of norepinephrine (0.02–0.05 μg/kg/min) was initiated. Additional administration of dobutamine (2–4 μg/kg/min) and colloids was considered if necessary. The transfusion threshold for hemoglobin varied between 70 and 90 g/L. In most cases, a tracheostomy was performed and maintained to ensure that the patient’s airway was clear throughout and after the surgery. If a tracheostomy was not performed, patients were extubated using a staged extubation kit.

After surgery, all patients were monitored overnight in the postanesthetic care unit before transfer to the general ward. Patients were mobilized after 5 days according to the internally established protocol. As all reconstructions were performed in the oral cavity, nutrition was provided exclusively using nasal or percutaneous gastric tubes until wound healing was confirmed. An effort was made to maintain a systolic blood pressure above 100 mmHg to ensure adequate blood flow to the flap. In the event of a drop in blood pressure, an appropriate volume of crystalloid fluids (250–500 mL) was administered. For signs suggesting overhydration, such as dyspnea, edema, or weight gain, furosemide was administered intravenously.

Postoperative flap monitoring included Doppler ultrasonography, whereby the site to be examined was marked intraoperatively using a suture marker. Clinical parameters, such as the color of the flap, were reassessed in ambiguous cases using the pinprick test. This was particularly relevant for patients with dark skin color, where “deepithelialisation” could also serve as an additional assessment criterion in situations requiring clarification. If any signs suggesting compromised blood supply to the flap were present, an immediate surgical revision was deemed necessary.

### 2.6. Statistical Analysis

**Figure 1:**
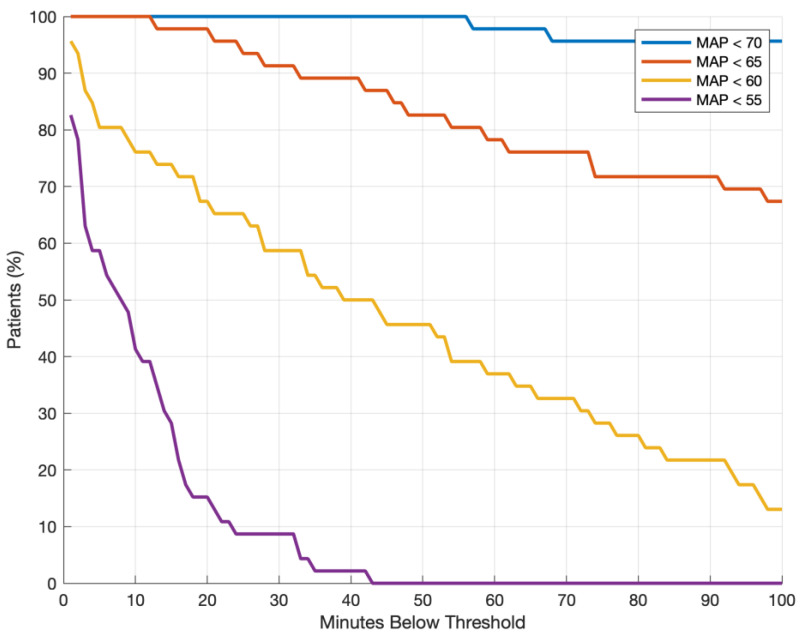
Proportion of patients with hypotension (MAP below a given threshold) and cumulative minutes under threshold. MAP: mean arterial pressure.

Data are expressed as counts with percentages or a median with interquartile ranges. Minutes below threshold were calculated per patient (Figure 1). Bivariable logistic regression models with flap-related complications (Table 1) and systemic complications (Table 2) as the predicted variables were used to assess the association of available baseline characteristics, including minutes below a given MAP threshold, with these outcomes. Variables with a *p*-value below 0.1 were then entered into multivariable logistic regression models, and MAP below a given threshold was added to this model to test whether a significant association could be found in multivariable regression models. A two-tailed *p*-value < 0.05 was considered statistically significant. MatLab (R2023a, Natwick, MA, USA) was used for data and statistical analyses.

## 3. Results

### 3.1. Population

In this study, a total of 46 adult patients participated, comprising 30 males (62.2%) and 16 females (34.8%). The specific baseline and intraoperative parameters can be found in Table 1 and Table 2, respectively.

### 3.2. Complications

Relevant systemic complications manifested in 10 out of 46 patients (21.7%), comprising four cases of cardiac decompensation, in one of which the patient died during hospitalization. Additionally, one case of pulmonary embolism (1/46, 2.2%), three cases of pneumonia (3/46, 6.5%), and five cases of refeeding syndrome (5/46, 10.8%) were reported. It is noteworthy that multiple complications could occur within a single patient.

Early surgical revisions due to flap-related complications occurred in 10 out of 46 patients (21.7%), stemming from issues such as anastomosis (1/46, 2.2%), bleeding (2/46, 4.3%), infection (2/46, 4.3%), and dehiscence/necrosis of parts of or the entire flap (5/46, 10.8%). All flap-specific complications that necessitated surgical interventions in the operating theatre were performed under general anesthesia. These interventions included the cessation of hemorrhage or the removal of hematoma (2/10), the revision of anastomoses (1/10), and the addressing of wound infections through opening and drainage (2/10). Additionally, flap dehiscence was assessed and treated by employing either an open or closed approach based on the extent of the dehiscence (4/10).

Within the first 24 h, there was one instance of surgical revision, involving a revision for bleeding and vessel repair due to vein rupture in the anastomotic area, thereby ensuring the flap’s survival. Subsequently, another case underwent revision surgery after 48 h due to anemic bleeding from the carotid artery and anastomotic insufficiency, resulting in the unfortunate loss of the flap and necessitating the harvest of an entirely new flap.

### 3.3. Intraoperative Hypotension

**Figure 2:**
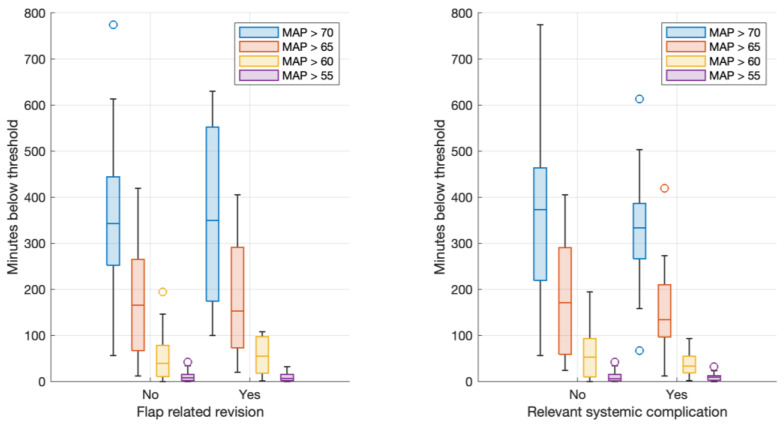
Boxplots of minutes below thresholds and flap-related revision (**left**). Boxplots of minutes below thresholds and systemic complications (**right**).

In bivariable logistic regression, there was no significant association between intraoperative hypotension (of any given threshold) and flap-related outcomes (Table 1, Figure 2). Fluid balance was more positive when flap revisions occurred (3692 mL (3101–4388) vs. 4859 (3555–6216), *p* = 0.026), whereas the parameters of fluid substitution and intraoperative blood loss did not show significant values (Table 1). 

The higher continuous administration of dobutamine was associated with significantly higher rates of revisions (total amount 27.5 mg (0–47.5) vs. 62 (38–109), *p* = 0.019), whereas the administration of norepinephrine was not (375.5 mg (0–989) vs. 312.5 (77–641), *p* = 0.493). Bivariable regression models identified intraoperative fluid balance (OR 1.9 (1.06 to 3.42) per 1 L increase in fluid balance, *p* = 0.0.026) and total dobutamine dose (OR 1.02 (1.00 to 1.05) per change of 1 mg of dobutamine, *p* = 0.019) as possible predictors of flap-related outcomes. This translated into a median difference of 62 vs. 27.5 mg of dobutamine between patients with and without flap-related complications, and a difference in positive fluid balance of 4.9 vs. 3.7 L between these groups. Other variables, including intraoperative blood loss and total fluid substitution, did not show a significant association. None of the assessed variables showed any significant association with systemic-related outcomes (Table 2).

### 3.4. Dobutamine

Total dobutamine dose as well as intraoperative fluid balance were then added to a multivariable regression to predict flap-related complications (Table 3). 

**Figure 3:**
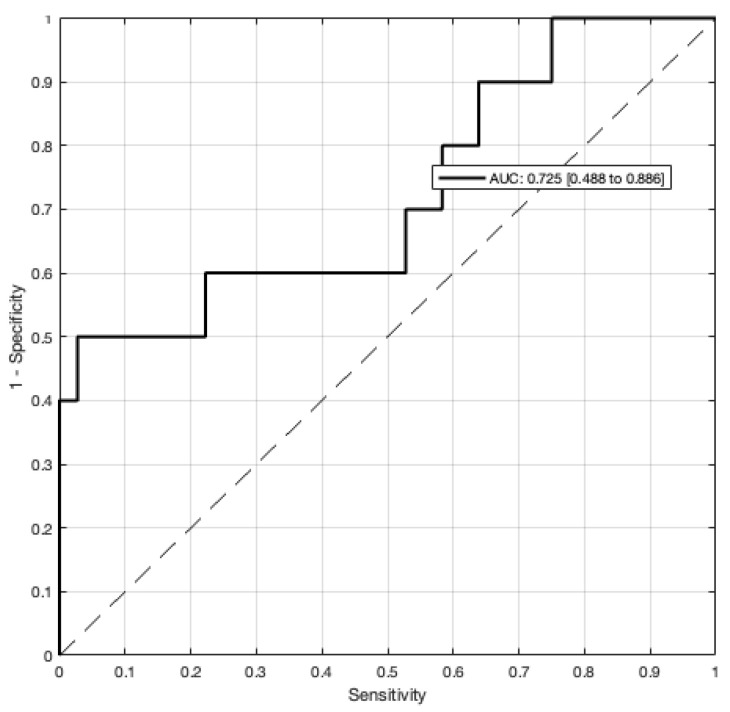
Goodness of fit using the area under the receiving operator curve (AUC-ROC) for a multivariable prediction model of flap-related revision (Table 3).

The model had a high goodness of fit, albeit with large confidence intervals (Figure 3). The included variables (total dobutamine dose and cumulative positive fluid balance) failed to reach significance in this model. None of the hypotension thresholds showed any significant association with flap-related complications when added to the multivariable model (MAP < 70 mmHg, *p* = 0.344; MAP < 65 mmHg, *p* = 0.481; MAP < 60 mmHg, *p* = 0.697; MAP < 55 mmHg, *p* = 0.366). 

## 4. Discussion

In a consecutive series of 46 patients undergoing major oncological head and neck surgery with fibula free flap head and neck reconstruction, we could not find an association between episodes in minutes underneath any threshold of hypotension with the risk of flap-related surgical revision. However, we found that in patients with flap revision, the intraoperative fluid balance was significantly more elevated, and these patients received a higher administration of dobutamine compared to the duration of the surgery and intraoperative blood loss. In addition, we found no correlation between the occurrence of cardiac and pulmonary complications and the duration of hypotension intraoperatively and during the early postoperative phase on the PACU.

In the existing literature, extended periods of hypotension have been shown to correlate with unfavorable outcomes not only in transplant surgery, but also in noncardiac surgical procedures. Hypotension is typically defined as a decrease in blood pressure below a MAP of 65 mmHg for a significant duration, with blood pressure fluctuations exceeding 25% of the initial reference value being linked to a higher incidence of adverse outcomes [5,12,16,17,18,21].

Kass et al. proposed that monitoring the cumulative proportion of intraoperative time with a mean arterial pressure (MAP) below 60 mmHg could be a valuable predictor of unfavorable postoperative outcomes and increase clinicians’ vigilance regarding possible adverse flap events in the postoperative period. The identification of flap failure as a consequence of intraoperative hypotension suggests a potential link between severe hypotension and the need for vasopressor administration. While few studies have reported flap loss in the presence of persistent perioperative hypotension [5,22], our findings indicate that short-term intraoperative blood pressure fluctuations might not play a significant role. However, our study challenges this conventional understanding. Surprisingly, we found that extended episodes of low blood pressure and cumulative hypotension do not seem to increase the risk of flap failure and the occurrence of systemic complications in this retrospective study. This is consistent with a recently published meta-analysis of randomized trials, showing that an intraoperative MAP of ≤60 mmHg was not associated with increased mortality [23]. In contrast, some of the actions taken in response to hypotension, such as administering higher volumes of crystalloids or adding inotropes, may potentially increase overall flap failure risk.

On the other hand, the administration of norepinephrine did not correlate with the risk of flap failure. In addition, the higher total dosages of dobutamine administered intraoperatively could be an illustration of a correction of impaired hemodynamics. In clinical practice, precise fluid administration and the avoidance of vasopressors during free tissue transfers often lead to recurrent episodes of low blood pressure in patients [24]. As a result, it has become common practice to use vasopressors to maintain mean arterial pressure and perfusion [22,25]. This approach helps to minimize the necessity for excessive fluid administration, which is a practice that has been linked to increased surgical and systemic complications [3]. A recent study in a similar cohort of patients identified diuretics as a potential marker of fluid overload, and low hemoglobin and intraoperative fluid as potential markers of flap-related complications [3]. In line with our findings in the current dataset, norepinephrine did not have any association with this outcome.

Inotropes are often administered as an adjunctive therapy to counter intraoperative hypotension, while norepinephrine is only administered in small amounts with the rationale that vasoconstriction may impair flap function. Recent data and our own findings imply otherwise, and within our dataset, there appears to be an indication that increasing dobutamine administration may be harmful. Furthermore, our in-house protocol may have led to potential fluid overload, which may further worsen outcomes; dobutamine was only administered after fluid loading had been attempted to restore normal blood pressure values. As our results show, intraoperative hypotension may not be a major determinant of flap failure and norepinephrine was not associated with adverse outcomes; this strategy should be revaluated. Other authors have also noted similar results and reported specific complications resulting from excessive fluid loading; however, in contrast to our own findings, low postoperative perfusion pressure was a predictor of flap failure [24].

The main goal of any hemodynamic intervention should be to restore normal organ perfusion. Cardiovascular function may be altered by fluid administration, vasoconstriction, and inotropic drugs. Recognizing the underlying reason for cardiovascular impairment seems to be of paramount importance in order to apply the correct strategy. If impaired cardiac function leads to hypoperfusion, this should clearly be treated accordingly, but if the main driver of hypotension is vasodilation through an administration of anesthetic agents, a strategy restoring normal blood pressure through vasoconstriction may be a safe and physiologically reasonable approach. A rational administration of norepinephrine to counteract anesthetic-induced vasodilation would be justifiable.

Our results underscore the critical role of vasopressors in free flap reconstruction, particularly in cases of severe hypotension that cannot be addressed solely through fluid administration. Some studies, including randomized controlled trials, indicate that the postoperative application of vasopressors can potentially enhance flap perfusion without causing harm, as demonstrated in research involving patients undergoing surgery to create radial artery forearm flaps [26]. This suggests potential advantages of vasopressor administration in specific scenarios [27]. Our study highlights the complex relationship between hypotension, vasopressor use, and flap outcomes in microvascular surgeries, emphasizing the need for personalized approaches in patient care.

The limitations of this study are the retrospective character and the relatively low number of included patients, although we were able to include 46 similar cases with a standardized surgical technique. It is important to acknowledge that undertaking a prospective, randomized study to address this question involves ethical challenges.

## 5. Conclusions

Our study indicates a correlation between severe hypotension and a bivariable association between fluid overload and dobutamine administration with a higher occurrence of flap-specific complications. Strategies that improve blood pressure should be aimed at the underlying pathophysiological process and inotropes should only be considered in states of impaired cardiac contractility. Assuming adequate cardiac function, an early administration of vasopressors in head and neck reconstructions appears to be safe and should be encouraged. Novel methodologies like prehabilitation and postoperative near-infrared spectroscopy of the flap offer a promise of enhanced outcomes in the future.

## Figures and Tables

**Table 1:** Baseline characteristics and intraoperative parameters for flap revision surgery.

	Reference	Flap Revision	Flap Revision	*p*-Value
	All	No	Yes	
	*n*, (%)	*n*, (%)	*n*, (%)	
Age, mean (SD), y	61.8 (55.3–67)	64 (55.8–68.4)	58 (55.3–64.1)	0.6
Sex				
Female	16 (34.8)	14 (38.9)	2 (20)	0.278
Male	30 (55.2)	22 (51.1)	8 (80)	
Alcohol use (yes)	24 (52.2)	17 (47.2)	7 (70)	0.211
Tobacco use (yes)	143 (77.7)	23 (63.9)	7 (70)	0.72
Arterial hypertension (yes)	15 (32.6)	11 (30.6)	4 (40)	0.574
Chronic obstructive pulmonary disease (yes)	7 (15.2)	5 (13.9)	2 (20)	0.636
Coronary heart disease (yes)	3 (6.5%)	2 (5.6)	1 (10)	0.62
Diabetes (yes)	3 (6.5%)	2 (5.6)	1 (10)	0.62
Weight (SD), kg	68.1 (61–80.4)	66.8 (59.5–79.2)	77 (68–83)	0.217
Preoperative radiotherapy (yes)	31 (67.4)	23 (63.9)	8 (80)	0.345
Renal insufficiency (yes)	2 (4.3)	1 (2.8)	1 (10)	0.353
				
Length of hospital stay (days)	16.5 (13–20)	16 (13–20)	18 (14–29)	0.057
Duration of surgery (min)	611.5 (540–720)	602 (538–682)	631 (589–743)	0.437
Intraoperative fluid balance (mL)	3726.5 (3130–4731)	3692 (3101–4388)	4859 (3555–6216)	0.026
Intraoperative fluid IV (mL)	5988 (4774–7280.6)	5953 (4766–7077)	6902 (5469–8516)	0.19
Intraoperative blood loss (mL)	675 (500–1000)	700 (475–1000)	631 (500–1000)	0.628
Vasopressor use				
Norepinephrine (total in µg)	375.5 (0–857)	375.5 (0–989)	312.5 (77–641)	0.493
Dobutamine (total in mg)	37 (0–59)	27.5 (0–47.5)	62 (38–109)	0.019
Postoperative nadir hemoglobin (total in g/L)	93 (85–100)	94 (85–100)	89.5 (85–100)	0.632
Intraoperative hypotension				
MAP < 70 mmHg (min)	342.5 (246–448)	342.5 (252–444)	349 (174–552)	0.79
MAP < 65 mmHg (min)	165.5 (73–273)	165.5 (67–265)	152.5 (73–291)	0.865
MAP < 60 mmHg (min)	40.5 (12–80)	39 (10.5–78)	54.5 (18–97)	0.803
MAP < 55 mmHg (min)	7.5 (2 –15)	8 (2–15)	6.5 (2–15)	0.937

**Abbreviations:** g/L = gram per liter; IV = intravenous; min = minutes; mL = milliliter; mmHg = millimeter of mercury; SD = standard deviation; y = year; µg = microgram.

**Table 2:** Baseline characteristics and intraoperative parameters for systemic complications.

	Reference	Systemic Complications	Systemic Complications	*p*-Value
	All	No	Yes	
	*n*, (%)	*n*, (%)	*n*, (%)	
Age, mean (SD), y	61.8 (55.3–67)	61.8 (53.5–65.7)	66.2 (59–73.8)	0.6
Sex				
Female	16 (34.8)	12 (38.7)	4 (26.7)	0.424
Male	30 (55.2)	24 (61.3)	6 (73.3)	
Alcohol use (yes)	24 (52.2)	16 (51.6)	8 (53.3)	0.913
Tobacco use (yes)	143 (77.7)	22 (71)	8 (53.3)	0.243
Arterial hypertension (yes)	15 (32.6)	10 (32.3)	5 (33.3)	0.942
Chronic obstructive pulmonary disease (yes)	7 (15.2)	4 (12.9)	3 (20)	0.533
Coronary heart disease (yes)	3 (6.5%)	1 (3.2)	2 (13.3)	0.228
Diabetes (yes)	3 (6.5%)	2 (6.5)	1 (6.7)	0.978
Weight (SD), kg	68.1 (61–80.4)	68.1 (57.3–79.3)	68 (64.3–82.5)	0.289
Preoperative radiotherapy (yes)	31 (67.4)	23 (74.2)	8 (53.3)	0.163
Renal insufficiency (yes)	2 (4.3)	0 (0)	2 (13.3)	1
				
Length of hospital stay (days)	16.5 (13–20)	16 (13–20)	18 (15–26.5)	0.073
Duration of surgery (min)	611.5 (540–720)	607 (536.3–674.8)	619 (560.3–726)	0.541
Intraoperative fluid balance (mL)	3726.5 (3130–4731)	3741 (3087–4660)	3683 (3434–4652)	0.585
Intraoperative fluid IV (mL)	5988 (4774–7280.6)	5829 (4796–7318)	6902 (4792–7250)	0.452
Intraoperative blood loss (mL)	675 (500–1000)	700 (500–1000)	600 (500–1025)	0.628
Vasopressor use				
Norepinephrine (total in µg)	375.5 (0–857)	349 (0–818.3)	428 (103.3–883)	0.668
Dobutamine (total in mg)	37 (0–59)	37 (0–55.8)	35.8 (2.5–75.5)	0.433
Postoperative nadir hemoglobin (total in g/L)	93 (85–100)	94 (87–100)	92 (83.3–100)	0.454
Intraoperative hypotension				
MAP < 70 mmHg (min)	342.5 (246–448)	373 (219–463.8)	333 (266.3–386)	0.559
MAP < 65 mmHg (min)	165.5 (73–273)	171 (58.8–290)	134 (96.5–209.8)	0.396
MAP < 60 mmHg (min)	40.5 (12–80)	53 (9.8–92.8)	33 (18.5–54.5)	0.211
MAP < 55 mmHg (min)	7.5 (2–15)	8 (1.3–15)	9 (2.3–12.8)	0.936

**Abbreviations:** g/L = gram per liter; IV = intravenous; min = minutes; mL = milliliter; mmHg = millimeter of mercury; SD = standard deviation; y = year; µg = microgram.

**Table 3:** Multivariable regression model to predict flap-related complications.

	OR	*p*-Value
Dobutamine (increase per 1 mg)	1.02 (1.00 to 1.04)	0.131
Intraoperative fluid balance (increase per 1 L)	1.61 (0.85 to 3.03)	0.093

## Data Availability

Data are unavailable due to privacy or ethical restrictions.
